# Disabling tinnitus and third nerve palsy following pontine hemorrhage: Application of ICF framework

**DOI:** 10.1002/ccr3.8405

**Published:** 2024-01-30

**Authors:** Vijaya Prakash Krishnan Muthaiah, Ignacio Novoa Cornejo, Sabarish Hariharan, Murugaraj Thyagarajan, Krishnamoorthy Gunasekaran

**Affiliations:** ^1^ Department of Rehabilitation Sciences School of Public Health and Health Professions State University of New York at Buffalo Buffalo New York USA; ^2^ Department of Physiotherapy Mahatma Gandhi Medical College and Research Institute Sri Balaji Vidyapeeth (Deemed to be University) Puducherry India; ^3^ Department of Medical Biochemistry College of Health Sciences Dambi Dollo University Dambi Dollo Ethiopia

**Keywords:** ICF framework, third nerve palsy, tinnitus

## Abstract

Spontaneous intracerebral hemorrhage commonly affects the brainstem. This report describes a 31‐year‐old male with hypertension who developed a pontine hemorrhage. The neurological deficits included left‐third nerve palsy, right‐sided weakness, and disabling tinnitus. Tinnitus is linked to central auditory pathway disruption. Magnetic resonance imaging revealed the hemorrhagic lesion and additional micro‐hemorrhages. The International Classification of Functioning, Disability, and Health (ICF) enhances rehabilitation by characterizing multifaceted stroke disability. The ICF profile revealed impairments in body structures/functions, limitations in activities/participation, and positive/negative environmental factors. ICF‐based goal‐setting informed interventions, including tinnitus retraining and physical/occupational therapy. Comprehensive ICF assessment is crucial for optimized, patient‐centered post‐stroke rehabilitation as it determines the extent of impact on functional level of the patient irrespective of disease severity.

## Graphical abstract

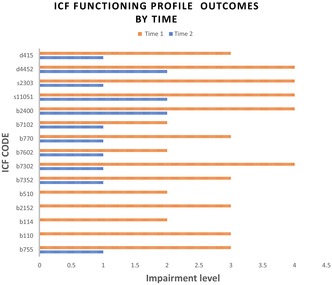

## 1 INTRODUCTION

Spontaneous intracerebral hemorrhage (ICH) accounts for 10%–20% of strokes worldwide, with hypertensive vasculopathy being the most common underlying cause.^1^ Brainstem hemorrhages represent one‐third of ICH and often arise in the pons.^2^ Vital sensorimotor pathways and cranial nerve nuclei traverse this region. Thus, characteristic signs of pontine ICH include hemiparesis, cranial nerve palsies, and impaired consciousness.^3^

Oculomotor nerve involvement causes ipsilateral ptosis and pupillary dilation with preservation of extraocular movements due to sparing of the superior branch.^4^ Abducens palsy and internuclear ophthalmoplegia reflect medial longitudinal fasciculus damage.^2^ Beyond focal deficits, brainstem hemorrhages frequently precipitate headaches and tinnitus due to vascular irritation of pain and auditory pathways.^5^ Tinnitus is a phantom perception of sound without any external acoustic stimulus. Persistent, troublesome tinnitus impairs concentration and quality of life.^6^ The National Institute on Deafness and Communication Disorders estimates that 10% of US population is suffering from some form of Tinnitus. Tinnitus can be of several types such as subjective, objective, pulsatile, unilateral, or bilateral.^7^, ^8^, ^9^

The International Classification of Functioning, Disability, and Health (ICF) codifies the multifactorial impacts of health conditions like stroke.^10^ The ICF enhances rehabilitation by elucidating specific limitations in body structures/functions, activities, participation, and environmental interactions. ICF‐based assessment informs goal setting and interventions to optimize functioning and societal participation.

This report presents an ICF profile of a patient with tinnitus and oculomotor palsy following pontine hemorrhage. MRI confirmed the hemorrhagic lesion. ICF components were examined to capture the breadth of disability. Tinnitus management and multidisciplinary therapies were tailored accordingly to promote recovery.

## 2 CASE PRESENTATION

**FIGURE 1:**
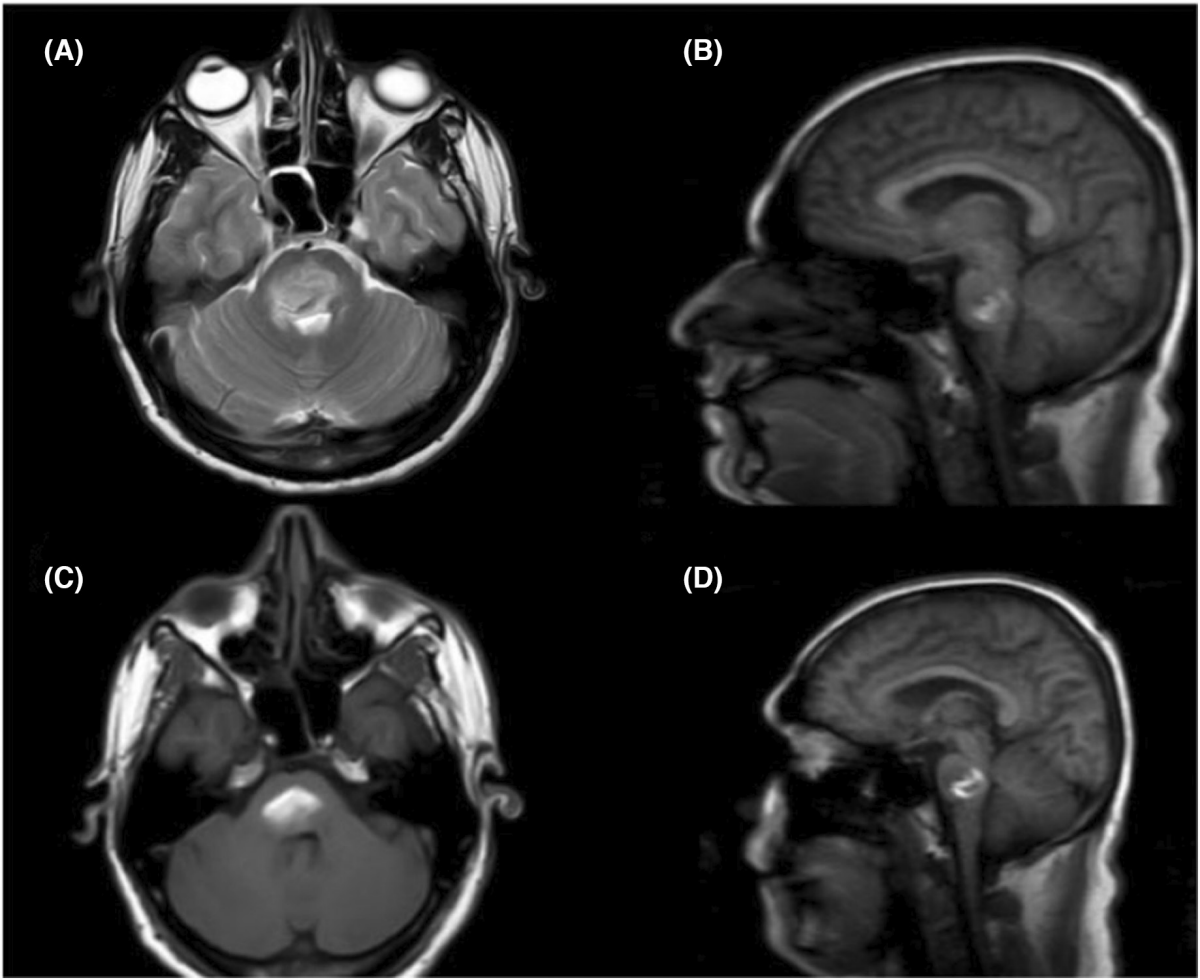
T1/T2 hyperintensities with adjacent perilesional edema are noted in pons extending to involve the right midbrain and superior part of the medulla oblongata, causing mild narrowing of the fourth ventricle. Areas of diffusion restriction and blooming were noted. Suspicious small feeding vessel noted‐ Few tiny foci of blooming noted in the bilateral lentiform nucleus and external capsule ‐ likely microhemorrhages. Mild periventricular T2/FLAIR hyperintensities noted ‐ likely vessel ischemic changes. Profile based on ICF Core Components.

A 31‐year‐old male with untreated hypertension presented with right‐sided involuntary movements followed by disorientation and impaired consciousness with a GCS scale of 12. Examination revealed left ptosis, anisocoria, right hemiparesis, and extensor plantar response. Brain MRI revealed a hemorrhage in the right pontine tegmentum with extension into the midbrain and medulla (Figure 1) and the differential diagnosis of Cavernoma bleeding was ruled out by a radiologist.

The neurological deficits indicated a left‐third nerve palsy and proper corticospinal tract involvement. During the hospitalization, the patient developed constant high‐pitched ringing in both ears. Tinnitus onset coincided temporally with the hemorrhage, implicating vascular disruption of central auditory pathways as the likely cause. He also experienced a persistent headache attributed to irritation of pain‐modulating brainstem nuclei by the hemorrhage.

**FIGURE 2:**
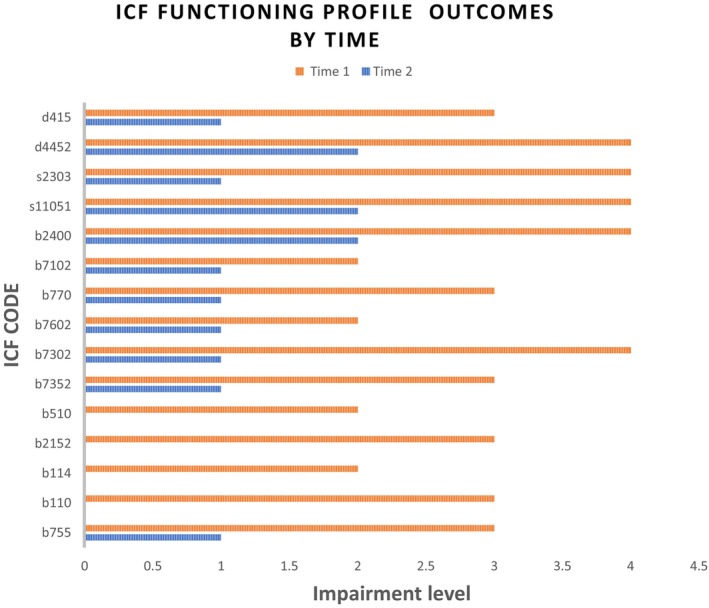
ICF functioning profile outcomes by time 1(orange) compared with time 2 (blue) b755 Involuntary movement reaction functions, b110 Consciousness functions, b114 Orientation functions, b2152 Functions of external muscles of the eye, b510 Ingestion functions, b7352 Muscle tone functions, b7302 Power of muscles of one side of the body, b7602 Coordination of voluntary movements, b770 Gait pattern functions, b7102 Mobility of joints generalized, b2400 ringing in ears or tinnitus, s11051 Pons, s2303 External ocular muscles, d4452 Reaching, d415 Maintaining a body position.

An ICF profile was created to characterize the myriad sequelae (Table 1, Figure 2). Impaired body structures and functions include the oculomotor nerve, corticospinal tracts, and central auditory system. Activity limitations comprised impaired vision, communication, self‐care, and mobility. Tinnitus hindered concentration and sleep. Environmental factors like social support and rehabilitation access were facilitators, while personal factors such as young age and hypertension control motivation enabled progress.

**TABLE 1:** ICF functioning profile: b: Body function; s: Body structures; d: Activity and participation; e: Environmental factors; prof: health professional or other professional; 0 no impairment; 1 mild impairment; 2 moderate impairment; 3 severe impairment; 4 = complete impairment; G: Intervention Goal; P = performance of…; C = capacity in. In environmental factors: +4: Complete facilitator; +3: Substantial facilitator; +2: Moderate facilitator; +1: Mild facilitator; 0: No barrier/facilitator; 1: Mild barrier; 2: moderate barrier; 3: Severe barrier; 4: Complete barrier. MD: Doctor of Medicine; PT: Physical therapy OT: Occupational Therapy; AuD: Audiologist.

Categories	Time 1: September 2022									Time 2: August 2023									G	Pr
	Impairment																			
Body functions: Physiological functions of body systems (including psychological functions	0		1		2	3		4		0		1		2	3		4			
b755 Involuntary movement reaction functions																			0	MD
b110 Consciousness functions																			0	MD
b114 Orientation functions																			0	MD
b2152 Functions of external muscles of the eye																			0	MD
b510 Ingestion functions																			0	MD
b7352 Muscle tone functions																			1	MD/PT
b7302 Power of muscles of one side of the body																			0	PT/OT
b7602 Coordination of voluntary movements																			0	PT/MD
b770 Gait pattern functions																			0	PT/OT
b7102 Mobility of joints generalized																			0	PT/OT
b2400 ringing in ears or tinnitus																			1	AuD
**Body structures**: Anatomical parts of the body such as organs, limbs, and their components	**0**		**1**		**2**	**3**		**4**		**0**		**1**		**2**	**3**		**4**		**G**	**Pr**
s11051 Pons																				
s2303 External ocular muscles																			1	MD
**Activities and participation:** Execution of a task or action by an individual and involvement in a life situation	**0**		**1**		**2**	**3**		**4**		**0**		**1**		**2**	**3**		**4**		**G**	**Pr**
d4452 Reaching																				
P																			0	PT
c																				
d415 Maintaining a body position																				
P																			0	PT/OT
c																				
**Environmental factors:** Make up the physical, social, and attitudinal environment in which people live and conduct their lives	**(+4)**	**(+3)**	**(+2)**	**(+1)**	**0**	**1**	**2**	**3**	**4**	**(+4)**	**(+3)**	**(+2)**	**(+1)**	**0**	**1**	**2**	**3**	**4**	**G**	**Pr**
e360 Others professionals																			(+4)	All

Specific management approaches were implemented based on the ICF profile. For tinnitus, the patient underwent tinnitus retraining therapy involving sound therapy and counseling. Vision rehabilitation and physical/occupational therapy also focused on facilitating activities and participation. The ICF profile will continue guiding multidisciplinary care during outpatient rehabilitation.

The patient was the biomedical diagnosis:

1. Acute cerebrovascular accident, hemorrhagic infarct in the right pons extending to the midbrain and bilateral medulla;
2. Systemic Hypertension;
3. Vascular headache secondary to brainstem hemorrhage;
4. Tinnitus;
5. Left third nerve palsy with horizontal gaze palsy;
6. Right‐eye exposure keratitis.

## 3 DISCUSSION

This patient's disabling tinnitus (b2400) and third nerve palsy (b2152) resulted from a hypertensive pontine hemorrhage. The ICF framework enhanced understanding of the myriad neurological impacts. The brainstem houses vital sensorimotor infrastructure, including cranial nerve nuclei (s11051), corticospinal/corticobulbar tracts, and auditory/oculomotor pathways.^2^ Accordingly, lesions cause deficits referable to involved structures.

The Oculomotor nerve tract was segregated into segments such as nucleus, fascicles, sub‐archnoid cistern, Cavernous sinus, and intra‐orbital segments.^11^ Oculomotor nerve fascicles originate from midbrain subnuclei before coalescing to exit the interpeduncular cistern.^4^ Though all segments of the nerve cannot be fully ascertained to the etiology, the involvement of partial third nerve palsy is confirmed with ptosis and mydriasis but spared extraocular movements. Medial longitudinal fasciculus damage produced a horizontal gaze palsy. Corticospinal tract involvement caused contralateral hemiparesis.

Tinnitus was a significant source of disability not directly attributable to focal damage. Instead, it likely arose from vascular irritation of central auditory networks.^5^ Similar central mechanisms induce headaches through the stimulation of pontine pain modulators. A disequilibrium in the pontine energy metabolism is attributed to the migraine pathophysiology,^12^ The headache satisfied the diagnostic criteria of C (localized in accordance with the site of hemorrhage) and D (unresolved) and was attributed to the non‐traumatic intracerebral hemorrhage. Refractory tinnitus and headaches represent major complications impacting the quality of life after brainstem hemorrhages.

Applying the ICF framework enabled a granular, patient‐centered characterization of health‐related domains. Beyond documenting neurological impairments, the ICF profile revealed resultant limitations in diverse activities and variable environmental influences two times, directly after the stroke; this could help follow the progress, obtain a prognosis, and make decisions according to future interventions. This understanding guided interventions targeting specific participation goals. Ongoing use of the standardized ICF language facilitates clear multidisciplinary communication and goal‐driven rehabilitation.^10^

Finally, the MRI was instrumental in precisely localizing the hemorrhage to the right pontine tegmentum. The correlation between MRI and functional performance confirmed the neurological deficits while excluding competing etiologies. Neuroimaging improves diagnostic accuracy and prognostication in ICH. However, optimal outcomes require complementing imaging with holistic frameworks like ICF that capture lived experiences.

## 4 CONCLUSION

This case exemplifies multifaceted disability following pontine hemorrhage. The patient exhibited left‐third nerve palsy and right hemiparesis from damage to the relevant midbrain and corticospinal tracts. Additionally, he developed centralized tinnitus and headaches due to vascular irritation of auditory and pain pathways. These complications added significant disability not explained by structural injury alone. MRI precisely localized the hemorrhage and elucidated the neurological manifestations.

A large‐scale evaluation of the ICF framework (b‐functions, s‐structures, d‐ activities and participations, and e‐environmental factors) on stroke patients indicated that stroke assessment using ICF is compatible with other common clinical scales.^13^ The ICF framework can be further simplified to include dichotomous responses which can be further evaluated by rasch analysis.^14^

Comprehensive application of the ICF framework codified impairments in body structures/functions, limitations in activities/participation, and the impacts of environmental/personal factors. This understanding informed interventions, including tinnitus retraining therapy and physical/occupational rehabilitation, to address discrete participation goals. Ongoing use of the ICF structure and terminology will optimize multidisciplinary care. Neuroimaging and holistic paradigms like ICF are essential for accurate diagnosis and patient‐centered management following turning off brainstem hemorrhages.

## AUTHOR CONTRIBUTIONS

**Vijaya Prakash Krishnan Muthaiah:** Conceptualization; supervision; writing – review and editing. **Ignacio Novoa Cornejo:** Writing – original draft. **Sabarish Hariharan:** Methodology; resources; writing – original draft. **Murugaraj Thyagarajan:** Validation. **Krishnamoorthy Gunasekaran:** Formal analysis; validation; writing – review and editing.

## FUNDING INFORMATION

The study is unfunded.

## CONFLICT OF INTEREST STATEMENT

The authors listed in this manuscript certify that they have NO affiliations with or involvement in any organization or entity with any financial or non‐financial interest in the subject matter or materials discussed in this manuscript.

## CONSENT STATEMENT

Written informed consent was obtained from the patient to publish this report in accordance with the journal's patient consent policy.

## Data Availability

The data supporting this study's findings are available on request from the corresponding author. The data is not publicly available due to privacy or ethical restrictions.
